# Patient focused registries can improve health, care, and science 

**DOI:** 10.1136/bmj.i3319

**Published:** 2016-07-01

**Authors:** Eugene C Nelson, Mary Dixon-Woods, Paul B Batalden, Karen Homa, Aricca D Van Citters, Tamara S Morgan, Elena Eftimovska, Elliott S Fisher, John Ovretveit, Wade Harrison, Cristin Lind, Staffan Lindblad

**Affiliations:** 1 Dartmouth Institute for Health Policy and Clinical Practice, Geisel School of Medicine, Dartmouth College, 1 Medical Center Drive, Lebanon, NH 03766, USA; 2 Institute of Public Health, University of Cambridge School of Clinical Medicine, Cambridge, UK; 3 Dartmouth-Hitchcock Health, Lebanon, NH, USA; 4 Medical Management Centre, Karolinska Institutet, Stockholm, Sweden; 5 Quality Register Center Stockholm, Karolinska Institutet and Stockholm County Council, Stockholm, Sweden

## Abstract

**Eugene Nelson and colleagues** call for registries of care data to be transformed into patient centred interactive learning systems

Large scale collection and analysis of data on patients’ experiences and outcomes have become staples of successful health systems worldwide. The systems go by various names—including registries, quality registries, clinical databases, clinical audits, and quality improvement programmes^1^ ^2^— but all collect standardised information on patients’ diagnoses, care processes, and outcomes, enabling systematic comparison and analysis across multiple sites. Hundreds of what we will term, for simplicity, “registries,” now exist around the world. The United Kingdom is home to over 50 clinical audit programmes,^3^ the United States has over 110 federally qualified registries certified to report quality metrics,^4^ and Sweden, perhaps the registry epicentre, has over 100, covering conditions from birth to frail old age.^5^

These registries have had far reaching effects. They facilitate public reporting, retrospective and prospective research, professional development, and service improvement. They reveal variations in practices, processes, and outcomes, and identify targets for improvement. In the UK, they have been associated with many notable successes, including improvements in management of cardiovascular disease and stroke,^6^ ^7^ cancer,^8^ and joint replacement.^9^

## Unmet potential

Nevertheless, few registries have realised their full potential. Feedback of data to participating clinical centres often lags well behind actual care, making data obsolete and less useful. Many registries have not caught up with the digital era, continuing to rely on manual data entry (and often double entry), which is tedious, expensive, and prone to error. The data may be restricted to a small number of uses, rather than being used for multiple purposes. Perhaps most problematic of all is that many registries have limited patient involvement in their design, oversight, or operations.^10^ Patients may not be asked to identify their priorities for data to be collected, so the information generated may only partly reflect what matters to them..^11^ Patients do not usually have access to the data collected (even when it is about them) or opportunities to add data outside medical encounters. This means they cannot use the data to support self management or shared decision making. 

Signs of change are, however, beginning to appear. The UK’s Healthcare Quality Improvement Partnership (HQIP) has an explicit patient and public involvement policy, and now includes patient representatives when developing specifications for its registries. National clinical audits in the UK, like those in Sweden, the Netherlands, and elsewhere,^12^ ^13^ are also beginning to incorporate patient reported outcomes alongside clinical measures.^14^

In the Netherlands, the Parkinson’s disease registry not only tracks strains on care givers as well as patient- and clinician-reported outcomes, it involves patients, families, physicians, and clinical scientists in developing guidelines to promote a consistently high standard of care.^12^ In the United States, the ImproveCareNow network for inflammatory bowel disease engages patients and families alongside care teams and scientists in its design, governance, and operation,^15^ enabling improved sensitivity to what matters to patients. Thus, though the registry had initially focused on measuring simple remission rates, partnering with patients revealed that patients and their families were more interested in prolonged, steroid-free remissions, which have improved from 55% to 78% in participating practices.^16^ The network has now added two UK sites (in Cambridge and London).

An especially exciting development is that some registries are gaining the capacity to collect data on patients’ priorities and support care in real time.^17^ The Swedish Rheumatology Quality Registry shows what can be achieved through this kind of patient centred approach. It enables patients to track symptoms at home to identify early signs of increased disease activity, supporting them to coproduce better care with their clinicians.^18^ The practices participating in the registry have documented a 50% decrease in inflammatory activity among people with rheumatoid arthritis.^19^

## Model for coproduction of health, healthcare improvement, and research

The growing emphasis on patient centredness in registries is consistent with the recognition of the importance of active partnerships between patients, clinicians, and health scientists to improve health, healthcare services, and research.^20^ ^21^ Patient centred registries could help realise the vision for a learning health system articulated by the US Institute of Medicine as one where “knowledge generation is so embedded into the core of the practice of medicine that it is a natural outgrowth and product of the healthcare delivery process and leads to continual improvement in care.” 22 In such a system, patient outcomes and experiences, as well as other valuable data, are continuously monitored and available in real time to both clinicians and patients to facilitate their joint work; importantly, the data are explicitly collected with a view to multiple uses and can be repurposed to support service improvement and scientific inquiry. 

Building on the structure and function of the registries described above, and the possibilities now offered by the rapid digitisation of healthcare, we have developed a generalisable model for a registry based, patient centred learning system for coproducing health improvement and research (box 1). A key feature of learning systems is that they are not simply a technical infrastructure: they are governed by strong values and a commitment to collective learning, not unlike Mintzberg and colleagues’ vision of a learning culture.^23^

**Box 1: Key features of registry based learning systems**

- Social network of patients and families encouraged to engage in the patient’s healthcare and supported by tools that enable them to track their health outcomes and support self care
- Collaborative network of clinical teams that can provide care and who engage in a system providing longitudinal and comparative data
- Sharing of power and responsibility among patients, clinicians, and scientists for designing, governing, and evaluating services, improvement, and research
- Digital collection and use of both clinical and patient reported outcomes to guide care and as a basis for improvement, research, and public health policy
- Demonstration of measurable improvement in individual and public health outcomes through improved adherence to current evidence and rigorous trials of new approaches
- Dissemination and translation of ideas and findings through publication in peer reviewed journals, presentations at meetings, and outreach to patients, clinicians, researchers, and health policy analysts

Our model brings together patients and families with clinicians and care teams to form a partnership (fig 1). Box 2 shows the main mechanisms supporting the system. One defining feature is that patients are able to share their perceptions of health, function, and wellbeing with their care team in real time; they can select measures that matter to them and enter their data outside clinical encounters, enabling them to monitor and continually assess their health. They can contribute to pre-visit planning, assist in monitoring treatment responses, and help to ensure that resources are being used for outcomes that truly matter to them.

**Figure f1:**
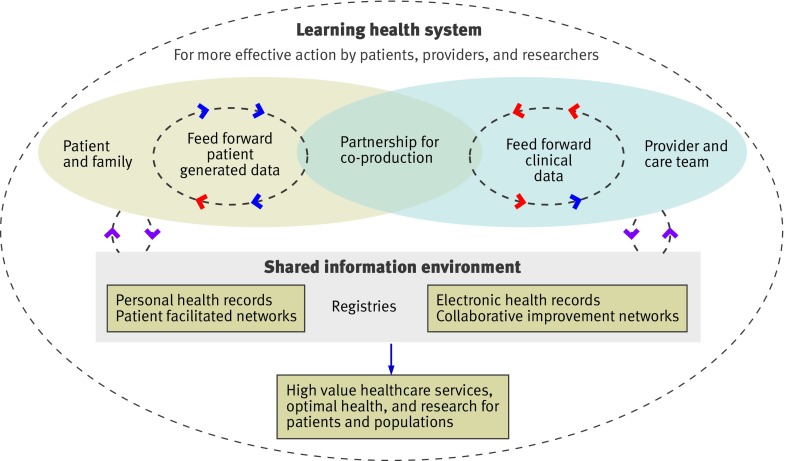
**Fig 1** Model of registry enabled care and learning health system

**Box 2: Key mechanisms used to build a registry based learning system**

*Data “feed forward” systems*—Patient reported and clinical data are continuously available both for patients and at the point of care for tracking health and planning care
*Decision support “dashboards”*—Graphs of patient level data over time enable patients and clinicians to detect relations between symptoms and interventions
*Reports for patients and clinicians*—Registry data are returned to both the patient and the clinician in meaningful summary reports that show trends over time. As a by product, registry databases can provide comparative data for practice improvement, research, and public reporting
*Patient and clinician facilitated networks*—Patient facilitated networks can foster social support and learning among patients with similar health conditions. Likewise, clinician facilitated networks can support the work of interdisciplinary care teams. Both types of networks ideally are co-curated by patients and professionals and provide information to support optimal health and high value care
*Multistakeholder engagement*—Collaborative networks including patients, clinicians, and researchers work together to share expertise, measurably improve outcomes and healthcare value, and conduct required research

Box 3 shows how the model works using the example of the Swedish rheumatology registry, which features consultations in which the patient and clinician sit together in front of a dashboard displaying the patient’s treatments and self reported outcomes as well as population based clinical, experiential, and functional data. The information supports patients and clinicians to become competent, confident, and equal partners who can share decisions.^25^ The dashboard is fed by a shared clinical database that is autopopulated by patient reported and clinical data that flow into and out of electronic and personal health records.

**Box 3: How the Swedish Rheumatology Quality Registry works**

**Shared care (http://www.youtube.com/watch?v=wjhkP8t1EmM)**

Karin Arvidsson is a middle aged professional woman who loves gardening. She developed rheumatoid arthritis several years ago and has taken four biological drugs that make her life much better. She manages her arthritis by “working with my doctor and my computer,” saying that being able to track her outcomes “helps me get through bad periods by myself.” Her trusted physician, Anita Domargard, says that Arvidsson is one of 25 000 Swedish patients in the national registry that tracks care using nationally agreed outcomes and allows her to “compare her results to the rest of Sweden.”

Domargard and Arvidsson sit together when they meet for a visit and view a dashboard that shows Arvidsson’s longitudinal outcomes (functional status, quality of life, joint counts, C reactive protein, etc) and her medications. They then decide which types of drugs are most likely to work best. Domargard says that she feels confident that the expensive drugs “are used in the best way.”

**Quality improvement (http://www.youtube.com/watch?v=wjhkP8t1EmM.)**

In 2008, Sven Tegmark became the director for rheumatology care for Gävle County, which provides services in four locations. His rheumatology programme had been participating in the registry for many years; unfortunately, the comparative results showed that the county’s patients had highly variable outcomes that were worse than the rest of Sweden.

Tegmark decided to use the registry to make two key improvements in the way care was delivered to Gävle’s rheumatology patients. Firstly, he encouraged all of the physicians to use the outcomes “dashboards” with every patient. Secondly, he developed a new delivery model that they call an “open-tight” system.^24^ In effect, patients were encouraged to use the dashboards at home to track outcomes and determine if they were in remission. If they were doing well, they were “open” to visit their physicians but making a visit was up to them. But if their outcomes suggested that they were out of remission, they were encouraged to make an appointment immediately and would be “tightly” cared for until they once again achieved remission. This helped make it possible for patients to be seen when they really needed to be seen and to use enlightened self management when they were doing well. The outcomes for Gävle County’s patients improved substantially after these changes (fig 2) such that they had better outcomes than those in the rest of Sweden.

**Figure f2:**
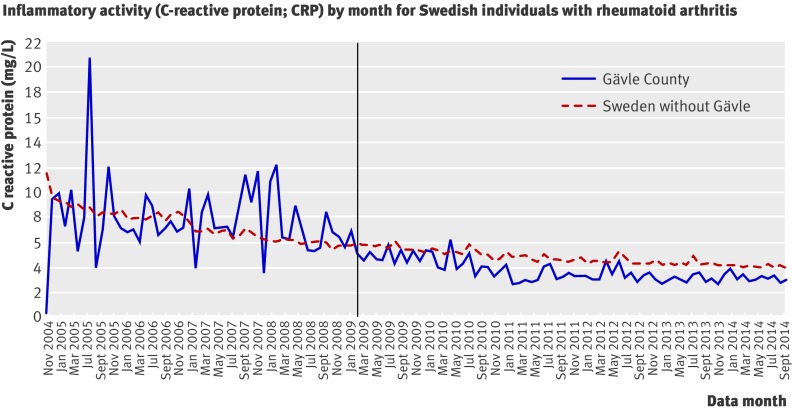
**Fig 2** Mean C reactive protein concentrations of patients in the Swedish Rheumatology Quality Registry living in Gävle County and the rest of Sweden

The functions and possibilities of a registry based learning system go well beyond the patient-clinical team dyad. The “small data” reported by patients and clinicians are recorded longitudinally in a secure registry platform. Configured appropriately, and with the right governance arrangements in place, this database can be used for many purposes: it creates opportunities for patients and families to get continuous real time access to peer and professional support using curated, facilitated networks^26^ and for clinicians to access collaborative improvement networks. It can be used to generate information to support activities to improve services, reducing the data burden for such activities and facilitating a more patient centred approach to improvement. Clearly, the database will be invaluable for many forms of research, including observational studies, n of 1 experiments, augmentation of results from randomised controlled trials, identification of participants for trials, and as the basis of studies where randomisation is neither appropriate nor practical.^27^ ^28^ Linking the database to other data sources, perhaps building on the Farr Institute for Health Informatics Research model (http://www.farrinstitute.org) of networking existing data sources into a larger, more data rich informatics structure, opens up new possibilities for patient centred research.

## Discussion

The increasing recognition that patients and their families should be involved in leadership of registries^29^ and the growth in technical capacities and methods of measurement offer enormous opportunities to bring together the interests and energies of patients and families, healthcare teams, and researchers.^30^ The role that patient centred registries could play in creating the conditions for a learning healthcare system within the NHS deserves careful consideration.^31^ ^32^ Founded on principles of coproduction, the model that we propose facilitates equal partnerships between patients and clinicians. The model intentionally aligns with the patient’s self-defined needs and priorities and refigures the opportunities for decision making. It is particularly relevant for people with chronic conditions, who rely on self monitoring and self management to achieve optimal health. The aim is to shift healthcare from a series of episodic encounters to a system in which everyone concerned about a person’s health—patients, families, clinicians, scientists, policy makers, and purchasers/commissioners—has the information they need for effective care, advancing knowledge, and improving services. 

The model does not, of course, promise a utopia. Some adverse consequences and risks can be anticipated, including those linked to the so called “quantified self,” where patients are seen as under surveillance and control (rather than empowered citizens).^33^ Innovators must keep in mind the risk that registry participants will focus too much on the numbers tracked by their fitness devices, rather than on the more complex goal of overall health.^33^ It will also be important to ensure that the model does not inadvertently create or exacerbate inequalities; some older people, for example, may continue to prefer paper based methods of data collection, and “big data” may create new forms of disadvantage. Issues of governance and regulation, including but not limited to the use of clinical data for research and other purposes, will need to be carefully worked through. Data security for registries will be critical, as will models of consent that are sufficiently agile to respect patients’ preferences and to cope with evolution of systems and changes in the purposes for which data may be used and in who may access and use which data over time.^34^ Although many problems will need to be overcome,^35^ successful registries indicate solutions are possible (box 4).

**Box 4: Addressing challenges for patient centred learning systems**

*Motivating participation—*Focus on important, measurable, and improvable health outcomes (eg, body mass index, remission rates)
*Organising, governing, and sharing power and influence—*Use community organising and peer production (eg, open source software development) principles and methods to start and manage the operation
*Finding ways to collect, display, and use “dashboards” into clinical workflows without adding extra work—*Employ trained coaches to upload existing data and then work with frontline clinicians and staff to adopt new work routines using the dashboard
*Empowering patients to make decisions and self manage—*Use the decision support dashboard as a catalyst for shared decision making and self management plans. Use a shared governance model (patients, clinicians, scientists) for planning, designing, and governing work
*Efficiently collecting valid patient reported and clinical data in busy practices across different electronic platforms—*Use a third party, internet based solution to extract selected clinical data elements from the electronic health record and the clinician, to collect self reported measures from patients using validated tools, and to instantly display all data in the dashboard. All of this can be done using secure, privacy protected processes
*Using data for learning, transparent reporting, and quality improvement—*Existing systems provide comparative, case-mix adjusted reports on variations in outcomes across practices, and encourage practice based improvements and adoption of best practices. They hold annual meetings to review performance, discuss lessons, and promote specific improvements
*Spreading and sustaining the system—*The Swedish registry covers the whole country and is funded from various sources, including government, foundations, and life science companies. ImproveCareNow has grown to include 80 paediatric specialty practices in just five years and is funded through membership fees

Registry based learning systems could unite patients, clinicians, and researchers to strive for, and ultimately coproduce, optimal health, high value services, and new knowledge that can be rapidly deployed to benefit individual patients and the public. Today’s registries have brought us a long way in improving healthcare; tomorrow’s registries, as patient centred learning systems, could bring us even further.

**Key messages**

Registries can evolve to become patient centred learning systems in which patients, clinicians, and scientists coproduce better health outcomes, improved services, and patient centred research
They can be used to make “dashboards” integrating patient reported and clinical data to support decisions about care
Registry data can be used to support practice based quality improvement, comparative benchmarking reports, and peer networks for clinicians and patients

## Notes

Contributors and sources: This article is based on a literature review and environmental scan that was conducted with support from the Robert Wood Johnson Foundation. ECN, PBB, EE, and SL have expertise in patient registries, clinical collaborative, and quality improvement; KH and CL have expertise in patient centred care; MD-W and JO have expertise in improvement and implementation science; ADVC and WH have expertise in formal literature reviews and programme evaluation; TSM and ESF have expertise in public policy, communications. and healthcare delivery science. ECN is the guarantor.
